# Why and how to engage expert stakeholders in ontology development: insights from social and behavioural sciences

**DOI:** 10.1186/s13326-021-00240-6

**Published:** 2021-03-23

**Authors:** Emma Norris, Janna Hastings, Marta M. Marques, Ailbhe N. Finnerty Mutlu, Silje Zink, Susan Michie

**Affiliations:** 1 Health Behaviour Change Research Group, Department of Health Sciences, Brunel University, Uxbridge, UK; 2 Centre for Behaviour Change, University College London, London, UK; 3 ADAPT SFI Research Centre & Trinity Centre for Practice and Healthcare Innovation, Trinity College, Dublin, Ireland; 4 Norwegian National Advisory Unit on Rehabiliation in Rheumatology, Diakonhjemmet Hospital, Oslo, Norway

**Keywords:** Stakeholders, Ontology development, Feedback, Social sciences

## Abstract

**Background:**

Incorporating the feedback of expert stakeholders in ontology development is important to ensure content is appropriate, comprehensive, meets community needs and is interoperable with other ontologies and classification systems. However, domain experts are often not formally engaged in ontology development, and there is little available guidance on how this involvement should best be conducted and managed. Social and behavioural science studies often involve expert feedback in the development of tools and classification systems but have had little engagement with ontology development. This paper aims to (i) demonstrate how expert feedback can enhance ontology development, and (ii) provide practical recommendations on how to conduct expert feedback in ontology development using methodologies from the social and behavioural sciences.

**Main body:**

Considerations for selecting methods for engaging stakeholders are presented. Mailing lists and issue trackers as existing methods used frequently in ontology development are discussed. Advisory boards and working groups, feedback tasks, consensus exercises, discussions and workshops are presented as potential methods from social and behavioural sciences to incorporate in ontology development.

**Conclusions:**

A variety of methods from the social and behavioural sciences exist to enable feedback from expert stakeholders in ontology development. Engaging domain experts in ontology development enables depth and clarity in ontology development, whilst also establishing advocates for an ontology upon its completion.

## Background

Ontologies allow us to specify entities and their relationships in a given domain [1]. Incorporating the knowledge of domain experts is essential for ensuring that the entities, definitions and relationships within an ontology capture the forefront of current knowledge [2]. Domain experts may be the instigators of ontology development, working to enable unification across diverse sources of knowledge in a given field. Alternatively, domain experts beyond the ontology development team may be recruited to provide a wealth of feedback to be incorporated into a given ontology. Engaging stakeholders with expertise in the domain of interest adds breadth and depth to ontology development and is more likely to ensure dissemination and engagement by the expert community [3].

Including a wide range of voices into ontology development is recommended by the Open Biological and Biomedical Ontology (OBO) Foundry: recognised as the gold-standard repository of interoperable ontologies in the scientific domain. OBO Foundry’s 10th principle of Commitment to Collaboration recommends that ontology content is scientifically sound (within the relevant domain) and appropriate for its intended use, meets community needs and supports interoperability with other ontologies or classification systems that experts may be developing or be aware of. However, the wording of this Principle is directed in particular towards collaborations between the developers of different ontologies, with the involvement of domain experts external to the development team not currently being a formal Foundry requirement. The Foundry’s 9th principle of Plurality of Users states that the ontology should be able to demonstrate that it is used by a plurality of different users, which is also close to involving expert stakeholders at the time of development. The OBO Foundry also has a resources hub in which best practices for ontology development are documented. Beyond ontology development best practices, Open Science principles advocate the prevention of research silos and widening the applicability of research [4]. Establishment of ontologies as freely accessible resources of knowledge also adhere to the FAIR data principles of being Findable, Accessible, Interoperable and Reusable [5].

Much guidance exists on ontology development and maintenance from a technical perspective, such as Building Ontologies with Basic Formal Ontology [1], the Introduction to Ontology Engineering [6] and Ontology Engineering [7]. There is also guidance on ontology reporting: Minimum Information for the Reporting of an Ontology (MIRO) [8], and ontology collaborative re-use: the Minimum Information to Reference an External Ontology Term (MIREOT) [9]. However, little guidance currently exists on how to conduct expert feedback, i.e. which method to use to enhance the ontology development process.

### Increasing development of ontologies within the social and behavioural sciences

A growing awareness of the need for ontology development is growing within the social and behavioural sciences (henceforth referred to as social sciences for brevity). Ontologies are beginning to be implemented in the social sciences to bring together complex areas of evidence [10], for example the OBO Foundry-registered Mental Functioning Ontology [11] and the Cooperation Databank ontology for cooperation research [12].

A key example of social sciences’ application of ontologies is in the field of behaviour change [10]. Bringing together disciplines such as psychology, sociology, economics and philosophy [13], behaviour change research works to improve the lives of humans across health, economics, environmental behaviours and beyond. Development of the behaviour change literature within these respective fields has resulted in overlapping terms for similar concepts, measures and methods [14]. This leads to ‘messy’ literature and a large degree of waste, as behaviour change research is difficult for researchers, practitioners and policy-makers to appraise, synthesise and implement [15–17].

Various projects are working to synthesise the growing knowledge base in behaviour change [18]. For example, the Science of Behaviour Change (SOBC) project is building a repository of measures used to assess self-regulation, stress reactivity and stress resilience, and interpersonal and social processes [19, 20]: synthesising validated measures in one central location. Over the last decade, taxonomies have been developed to group and specify terms in behaviour change, such as the Behaviour Change Techniques Taxonomy (BCTTv1) to specify the content of behaviour change interventions [21, 22].

Ontologies are also being developed to standardise and synthesise behaviour change, for example, the Behaviour Change Intervention Ontology being developed within the Human Behaviour-Change Project [23–25]. This ontology aims to represent all features within any behaviour change intervention evaluation report and is being used to guide data synthesis in an Artificial Intelligence system of published behaviour change intervention reports [24]. Associated with this project is a research program, aiming to better specify the plethora of behaviour change theories across disciplines, via graphical, computer-readable representations and formal specifications of terms and relationships [26, 27]. However amongst these early advances, ontology development in the social sciences is still relatively novel.

While the social sciences have much to learn in terms of computational ontology development, they have much to contribute in methods of engaging expert stakeholders to improve development and adoption of ontologies. Social sciences have a strong tradition of engaging stakeholders in co-production of intervention development [28] and in the development of standardised terminologies and reporting frameworks. For example, the Behaviour Change Techniques Taxonomy (BCTTv1) [21] was iteratively developed with expert feedback and consensus exercises. Component ontologies within the Behaviour Change Intervention Ontology have been developed with stakeholder feedback to ensure relevance [29, 30]. This paper aims to draw on social science research to: (i) demonstrate how expert feedback can enhance ontology development, and (ii) provide practical recommendations on how to conduct expert feedback in ontology development.

## Main text

### Considerations when selecting method(s) of expert stakeholder engagement

Various considerations are important when designing a protocol for engaging expert stakeholders. First, what type(s) of data will be most useful to the research group? A broad range of methods can be used to assess expert feedback for ontology development. At a simplistic level, the methods and results of engaging experts can be viewed in two main forms. Quantitative feedback, such as structured questionnaires with ratings (e.g. ‘On a scale of 1 to 5’…) may be useful for informing ontology coverage and structure. Qualitative feedback, such as comments elicited by interviews and open-ended questions within questionnaires [31] may be useful for detailed feedback and suggestions of alternative labels, relationships or definitions.

Second, what mode of delivery will be most feasible for the expert stakeholder group? Is it preferable in your context to do in-person expert meetings, providing easier facilitation of tangent discussions that may benefit ontology development? Or is online asynchronous or synchronous feedback and engagement preferable? Online methods have the advantage of increasing inclusion across geographical regions and of increasing a wide range of voices to be heard that in-person sessions don’t always enable [32]. The potential for online meetings that may last one or more days also allows for more reflection and informal discussion that may help to clarify and unify thinking.

Third, what amount of engagement will be most feasible for the expert stakeholder group? Consider how much time or resources experts will have to complete a review of the ontology being developed. If your ontology is extensive or if you require detailed feedback across entity labels, definitions and relationships, you may wish to divide feedback on different aspects of the ontology by different experts to reduce their time burden.

Finally, consider what data will be collected in your expert stakeholder feedback and how it will be stored. Check whether you require Institutional ethical approval for data collection. Data may include stakeholder names and other personal information, for which you may need to consult local IT support and a Data Protection Officer to ensure compliance within your geographical location, such as the European Union’s General Data Protection Regulation (GDPR) [33] law on data protection [34].

### Methods of engaging with expert stakeholders

#### 1. Existing practices using mailing lists and issue trackers

The OBO Foundry recommends collaborative ontology development with the involvement of both ontologists and domain experts, within its Commitment to Collaboration principle. The specific recommendations for *how* such involvement should be achieved, however, only refer to the specific online channels rather than the methodology involved in managing such processes.

In particular, the Foundry recommends that each ontology has an accompanying mailing list clearly listed alongside it, so that stakeholders and members of the public can join, as well as an issue tracker in which specific user requests and feedback can be received and tracked. In addition, there is a community-wide mailing list for general discussions between all the Foundry ontology developers and users (obo-discuss) and one for issues related to the shared common upper-level ontology BFO (bfo-discuss). Other community-wide ontology-related mailing lists outside of the OBO Foundry include the Protégé User mailing list and the ontolog forum. There are also region-specific ontology mailing lists (e.g. ontology-uk@googlegroups.com).

Issue trackers enable users to provide feedback on potential changes to a published ontology and also provide a log of resultant improvements. Such issue trackers are commonly run using GitHub, such as the Ontology of Biomedical Investigations (OBI), Environment Ontology (ENVO) and Gene Ontology (GO) issue trackers.

The advantage of using mailing lists and issue trackers for communication with members of the community and stakeholders is that such communication becomes part of the documentation associated with the ontology: all messages are archived and public, and thus a body of knowledge builds up over time that can be consulted and referred back to in the future. It acts as a useful broadcast medium for members of the community to notify the wider community of resources, tools, and events, and also to recruit participants for studies or surveys. For example, the development of the Minimum Information for the Reporting of an Ontology guidelines (MIRO) [8] included obtaining feedback from over 100 experts via an online survey, and participation in this survey was recruited via mailing lists such as the Protégé User list and the OBO Discuss list.

A challenge with mailing lists for getting feedback is that they tend to become dominated by discussions by a smaller number of people, while some interested persons may be afraid to reach out using such a public medium. Discussions on contested topics may become heated, particularly since ontologies need to represent the needs of a diverse range of stakeholders and their different needs and wishes may at times be opposed. Thus, there is a need for careful and sensitive ongoing mediation and moderation. Issue trackers on the other hand, while perhaps more discreet than community wide mailing lists, are quite a passive mechanism for obtaining feedback and tend to be used mainly for specific feedback and problem requests, rather than general feedback or feedback in advance of the development of a particular aspect of the ontology.

**Table 1:** Methods for expert stakeholder engagement in ontology development

Method	Description	Examples
**Advisory Board and Working Groups**	Small and select group of domain experts evaluate progress of ontology development and providing unbiased strategic and scientific recommendations. Delivered via in-person or online meetings, or by email	Exposure Ontology (EXO) [35] Human Phenotype Ontology (HPO) [36] Scientific Advisory Board of the Human Behaviour-Change Project [23]
**Expert Panels**		
**Feedback tasks**	Experts asked to provide feedback on the whole ontology or specified elements of it Delivered in-person or using online survey tools (e.g. Qualtrics, Limesurvey, Google Forms).	Development of the Behaviour Change Intervention ontology (BCIO) within the Human Behaviour-Change Project [29, 30, 37] *Related example from social and behavioural sciences*: Development of COVID-19 research priorities in psychology [38] Development of the Theories and Techniques Tool [39] Self-Determination Taxonomy [40]
**Consensus exercises**	Consists of two or more sequential rounds of questions and feedback to experts, aiming to achieve consensus. Delivered using online consensus (e.g. Loomio, DelphiManager) or survey tools (e.g. Qualtrics)	*Related examples from social and behavioural sciences*: Links between Behaviour Change Techniques and Mechanisms of Action [41] Delphi consensus to identify priorities for methodological research in behavioural trials in health research [42] Behaviour Change Techniques Taxonomy v1 [21]
**Discussions**	Experts are invited to engage in online discussions with other experts on a specific topic, e.g. relationships between certain ontology entities. Delivered using project websites or online consensus tools (e.g. Loomio, DelphiManager). It can be synchronous or asynchronous.	Cognitive Atlas [43] *Related examples from social and behavioural sciences*: Links between Behaviour Change Techniques and Mechanisms of Action [41]
**Workshops**	Small-group discussions and presentation of specific proposals related to ontology development. Decisions are taken by the group during the workshop. Delivered via in-person or online meetings	Computable Exposures Workshop [44] NCI Semantic Competency Query Review [45] Phenotype Ontologies Traversing All The Organisms (POTATO) workshop [46] Workshops at International Conference of Biomedical Ontologies (ICBO) Workshops at bi-annual Formal Ontologies in Information Systems *Related examples from social and behavioural sciences*: Current development of the E-cigarette ontology [47]

#### 2. Proposed additional methods drawing from social and behavioural sciences

In the context of the social and behavioural sciences, expert feedback can be divided into two overarching methods: (1) Advisory Boards and Working Groups; or (2) Expert Panels. Consulting with expert panels can be done through specific feedback tasks, consensus exercises, small group discussions and workshops. Table 1 provides a summary of methods to engage expert stakeholders in ontology development.

### Advisory boards and working groups

Advisory Boards and Working Groups involve a small and select group of domain experts evaluating progress of ontology development through (e-)meetings and/or e-mail. These groups are brought together to provide feedback at key points within a given project and are typically constituted by researchers and practitioners from disciplines that are relevant for the specific ontology. For example, the Exposure Ontology (EXO) [35] was developed by a working group of the ontology authors and 10 other scientists from academic research, regulatory, industrial, and nongovernment organisations. Each working group member was required to provide feedback on the comprehensiveness of the developing ontology and identify and annotate three additional manuscripts in their specific area of expertise using the ontology. This pilot annotation was evaluated by the group and used to make iterative refinements to the ontology. A second example is the Human Behaviour-Change Project (HBCP) with a Scientific Advisory board of 36 international experts across diverse fields including behavioural science, public health, computer science, ontologies and system architecture. Terms of Reference were established at the commencement of the Scientific Advisory board, with board members meeting online up to twice a year and asked to provide feedback on pre-circulated reports.

### Expert panels

Expert panels are larger groups of leading researchers or practitioners in the specific domain of the ontology that participate in structured tasks to evaluate the ontology under development. They provide structured feedback on characteristics of the ontology, such as its structure, clarity, utility, coverage or overlap with other ontologies or classification systems. Recruitment can be via project mailing lists, direct invitations or open calls for experts via social media. The number and size of the panel varies depending on the purpose of the consultation. An example of such methods used in social sciences includes the establishment of psychological science research priorities related to COVID-19 [38]. A core expert panel of nine psychologists generated and judged research priorities over 10 h-long online meetings, assisted by a wider expert panel of 16 psychological scientists across varying disciplines of psychology. The cumulative research priorities of this work were then verified by 539 psychologists contacted by snowball sampling via professional network mailing lists such as the British Psychological Society [38]. Within the Human Behaviour-Change Project, around 100 experts were contacted via email to evaluate each ontology. Of those invited, 23.5 % [30] to 64 % [29] opted in to provide feedback in subsequent tasks. Software commonly used for online expert panel tasks includes online consensus tools (e.g. Loomio, DelphiManager) and survey tools (e.g. Qualtrics, Google Forms or LimeSurvey).

#### - Online feedback tasks

Online feedback tasks involve consulting with a large pool of experts, usually internationally. An example of such methods used in social sciences is development of the Self-Determination Taxonomy, which was developed, reviewed and finalised with international expert feedback in a seven-step procedure involving group discussions, feedback and iterative development [40]. Crowd-sourcing methods and portals for content gathering can also be seen as a form of online feedback task and can also be made available to experts. For example, the Cognitive Atlas ontology [43] was developed via an online platform that allowed registered users to comment on existing classes and relationships as well as propose new ones. The Qeios platform for definition-enhanced open publishing is being used by the Addiction Ontology to enable expert review, comments and feedback on ontology definitions [48].

#### - Consensus exercises

Consensus exercises consist of two or more sequential rounds of questions and feedback to experts, aiming to achieve consensus across the group, such as in Delphi exercises [49] or using Nominal Group Technique [50]. This may involve an initial rating round to gather initial views, an evaluation round where experts are provided with everyone’s responses and a final rating round to develop a final consensus. Within ontology development, consensus exercises can be used to build consensus on an ontology’s coverage and remit, as well as on specific entities, their definitions and relationships [37].

An example of such methods used in social sciences include the Theories and Techniques Project, a Nominal Group Technique study where 105 international behaviour change experts iteratively rated, discussed and re-rated links between 61 commonly used Behaviour Change Techniques (BCTs) and 26 Mechanisms of Action (MoAs) [39, 41]. Another study used an international Delphi consensus to identify priorities for methodological research in behavioural trials in health research in 15 core members of the International Behavioural Trials Network [42].

#### - Online discussions

Experts are invited to engage in online discussions with other experts on a specific topic, e.g. relationships between certain ontology entities. Such discussions can be included as part of a wider consensus exercise, or as an independent online feedback task. Within ontology development, the Cognitive Atlas ontology was developed with discussions taking place within its website to drive curation of an ontology of cognitive science [43].

#### - Workshops

It is common practice currently within the OBO Foundry community and its Commitment to collaboration, for the development of ontologies to involve in-person development workshops with both domain experts and ontology expert attendees. The biomedical informatics community also has a long history of hands-on development of informatics resources using specific types of hands-on workshops called “hackathons” (for implementation of tools and resources) and “curation jamborees” (for the collective curation of data or knowledge resources), and ontologies have frequently been included in these sorts of workshops. For example, ontology development has been a part of the long-standing annual “BioHackathon” workshop [51]. However, a lack of pre-existing expertise in appropriate tools and methods can present a barrier to wider participation of domain experts – as opposed to ontology or informatics experts – in this kind of workshop. Workshops are a valuable method for gathering feedback as during workshops, specific proposals are discussed and strategic decisions are taken in the room. However, holding such workshops depends on the availability of dedicated funding, such as recent funding for ontology workshops awarded to the Monarch Initiative. As an alternative which does not require dedicated funding, workshops can be held alongside ontology conferences. For example, both the annual International Conference on Biomedical Ontologies and the biannual Formal Ontology in Information Systems conferences accept proposals of workshops to be held alongside the conference. During COVID-19 and beyond, these workshops are being moved to online alternatives, allowing timely decisions to be made and easier collaboration with international colleagues [52]. As yet, there are no dedicated ontology workshop tracks within relevant social science conferences.

The appropriate expert engagement method(s) to appraise a developing ontology depends on a variety of factors. While we provide here some guidance, each research team should select the criteria that fits their aims. We provide some questions to guide the selection of the method(s) in Table 2.

**Table 2:** Questions to guide selection of the expert engagement method

1. What is the research question you intend to answer with the expert activity?
2. What is the representativeness you aim to achieve in the expert activity?
3. What is the dedicated funding available?
4. To which resources (e.g. staff, time, online software, meeting room) do you have access?
5. What is the time-frame for your expert engagement activity?

#### 3. Collating and recruiting a pool of expert stakeholders

The expert stakeholders appropriate for the development of any given ontology will vary project-by-project. First, identify what skills, expertise and knowledge are required to evaluate the ontology. If international relevance is an aim for the ontology, ensure that your experts have adequate geographical stakeholder representation. If the ontology has a multidisciplinary remit, ensure that your experts have expertise from across these disciplines. The team can then narrow down potential stakeholders, and make sure they obtain the expertise needed.

Second, develop a recruitment strategy to identify experts. Recruitment can take place via direct invitations [35, 38] or via ‘snowball sampling’ of open requests to mailing lists, social media, key organisations or professional societies [37, 41]. Recipients to open requests can be filtered to ensure only individuals with specific expertise or amount of experience are included. For example within the Human Behaviour-Change Project, an open invitation to provide feedback on behaviour change ontologies required interested experts to complete an introductory filter Qualtrics questionnaire [37]. This included demographic information such as career level, discipline and country, as well as self-assessment of their experience designing, reporting and publishing behaviour change intervention reports and in features of behaviour change interventions for which ontologies were being developed. It is important to emphasise what the expert gets out of taking part, and how they might benefit from providing their time and expertise, such as acknowledgement or co-authorship in subsequent papers or presentations, or introduction to a wider professional network. It is also important to be realistic and honest about the time commitment. The development team must establish whether the stakeholders will be involved in a one-off task or feedback session, or whether the process is iterative, time consuming, and requires continuous commitment from experts.

#### 4. Analysing and reporting expert stakeholder engagement

A data analysis plan to specify how and who will be performing analysis of expert feedback should be developed prior to data collection. First, consider if one or multiple members of the ontology development team will be available to process this feedback. Alternatively, you may wish to recruit someone beyond the development team to review the feedback to minimise potential bias, such as an ontology expert without expertise in the domain being studied. Secondly, decide a protocol for the process of feedback analysis. Will you perform a point-by-point analysis of all individual comments, with all comments being equally valid? Or if feedback is non-anonymised, will you weight comments according to amount or area of expertise?

Data arising from expert feedback should be made as open as possible, to make any resultant changes made to the ontology transparent and facilitate replication [4]. Plans to make anonymised or non-anonymised feedback publicly available must be included in any prior ethics applications, incorporating data protection considerations [34]. The Open Science Framework is a popular repository for sharing data, materials and code arising from research. Within development of the Behaviour Change Intervention Ontology, anonymised expert comments and iterative versions of ontologies before and after feedback were made available on the project's Open Science Framework page [29, 30].

## Conclusions

Engaging domain experts is crucial for establishing comprehensive, accurate and clear ontologies, as well as benefiting dissemination and engagement by the expert community [3]. This discussion paper drew on social and behavioural science research to demonstrate the ways in which expert feedback can enhance ontology development, methods that can be used to engage experts and how to recruit and analyse expert feedback. We consider that there is no ‘one-size-fits-all’ approach to expert involvement. How, when, who and why experts should become involved will vary from ontology to ontology. We would propose external scrutiny by domain experts of all ontologies, to ensure their comprehensiveness, accuracy and relevance.

## Data Availability

No data or materials collected within this synthesis of published research.

## Abbreviations

BCIO: Behaviour Change Intervention Ontology
BCTTv1: Behaviour Change Techniques Taxonomy v1
BFO: Basic Formal Ontology
EXO: Exposure Ontology
GDPR: General Data Protection Regulation
HBCP: Human Behaviour-Change Project
MIREOT: Minimum Information to Reference an External Ontology Term
MIRO: Minimum Information for the Reporting of an Ontology
OBO: Open Biological and Biomedical Ontologies
SOBC: Science of Behaviour Change
